# The Complement System and ANCA Associated Vasculitis in the Era of Anti-Complement Drugs

**DOI:** 10.3389/fimmu.2022.926044

**Published:** 2022-06-23

**Authors:** Yasutaka Kimoto, Takahiko Horiuchi

**Affiliations:** Department of Rheumatology, Hematology and Metabolic Diseases, Kyushu University Beppu Hospital, Beppu, Japan

**Keywords:** complement, alternative pathway, ANCA, C5a, avacopan

## Abstract

ANCA (anti-neutrophil cytoplasmic antibody)-associated vasculitis (AAV) is the condition in which ANCA, as an autoantibody, is associated with the pathogenesis of vasculitis in small blood vessels, mainly in the ear, nose, throat, kidney, lung, and nerves. These diseases are important because they can be fatal due to renal failure and pulmonary hemorrhage if not promptly and appropriately treated. Recently accumulated evidence has shown that C5a produced by the complement alternative pathway primes neutrophils, which in turn activate the complement alternative pathway, leading to the pathogenesis of AAV. Avacopan (CCX168), a C5aR antagonist was shown to be effective against AAV, and it has been a novel therapeutic option, becoming a novel anti-complement drug to modulate inflammatory diseases.

## Introduction

ANCA (anti-neutrophil cytoplasmic antibody)-associated vasculitis (AAV) is the condition in which ANCA, as an autoantibody, is associated with the pathogenesis of vasculitis in small blood vessels, mainly in the ear, nose, throat, kidney, lung, and nerves (1). Microscopic polyangiitis (MPA), granulomatous polyangiitis (GPA), and eosinophilic granulomatous polyangiitis (EGPA) are classified as AAV. These diseases are important because they can be fatal due to renal failure and pulmonary hemorrhage if not promptly and appropriately treated (2, 3).

Renal manifestations include crescentic glomerulonephritis, but in contrast to glomerulonephritis caused by immune complex glomeruli or anti-glomerular basement membrane antibodies, AAV is classified as a so-called “pauci-immune” disease in which the renal glomeruli lack or have minimal immunoglobulin and complement deposition (4). Common laboratory tests such as plasma C3 and C4 are not decreased in most AAV patients. In addition, immune complexes are thought to cause tissue damage through the activation of the complement classical pathway. For these reasons, it has been thought that the complement system is unlikely to be involved in AAV.

On the other hand, recently accumulated evidence has shown that C5a produced by the complement alternative pathway primes neutrophils, which in turn activate the complement alternative pathway, leading to the pathogenesis of AAV. Avacopan (CCX168), a C5aR antagonist was shown to be effective against AAV, and it has been a novel therapeutic option, becoming a novel anti-complement drug to modulate inflammatory diseases.

## ANCA Associated Vasculitis (AAV)

AAV is a primary vasculitis classified as small vasculitis in the 2012 Revised International Chapel Hill Consensus Conference Nomenclature of Vasculitides and presents with necrotizing vasculitis mainly in small arterioles, capillaries, and small veins. Microscopic polyangiitis (MPA), granulomatous polyangiitis (GPA), and eosinophilic granulomatous polyangiitis (EGPA) are classified as AAV (4). Epidemiologic differences in incidence and clinical presentation are observed according to the environmental and genetic background of the patient. The cause is undetermined, but it is thought that genetic and environmental factors influence each other, resulting in the production of the autoantibody ANCA, which causes vasculitis. These diseases are characterized by the detection of autoantibodies against myeloperoxidase (MPO) and proteinase3 (PR3) in neutrophil cytoplasm at a high frequency. Patients with AAV develop systemic vasculitis and present with a variety of symptoms and organ dysfunction. Especially the kidneys and lungs, which are rich in small vessels, are the most frequently affected organs, and alveolar hemorrhage and RPGN are the most important prognostic factors.

The diagnosis of AAV is often based on the European Medicines Agency (EMA) classification criteria written by Watts (5), but new classification criteria for each AAV have been proposed by ACR/EULAR in 2022 (6–8).

In 2016, EULAR recommended the combination of cyclophosphamide (CY) or rituximab (RTX) in addition to glucocorticoid (GC) as first-line induction therapy for severe AAV. Patients with AAV without severe organ involvement may receive methotrexate (MTX) or mycophenolate mofetil (MMF) in addition to GC. After induction of remission, as maintenance therapy, azathioprine (AZA), RTX, MTX, or MMF for MPA/GPA and AZA for EGPA are used as immunosuppressive agents in addition to low-dose GC (9).

In patients with AAV, high cumulative doses of corticosteroids have been reported to be associated with several short- and long-term complications and comorbidities (10).

## The Complement System

The study of the complement system has a long history. In 1895, Bordet of the Pasteur Institute discovered a component in serum that had bacteriolytic activity. In addition to antibodies, which are heat stable, the bacteriolytic activity of serum requires a component that is inactivated by heating to 56°C for 20 minutes, which was named “complement”. Subsequent studies revealed that complement consists of multiple components, which were identified successively. The group of innate immune factors consists of more than 30 components, including plasma proteins, receptors on cell membranes, and regulatory factors. In addition, complement components have been identified not only in vertebrates other than mammals such as humans but also in sea anemones and other sponges lacking an acquired immune system, indicating that the complement system is a primitive immune mechanism that has been part of the evolutionary process of all living organisms since ancient times.

The complement pathway proceeds by degrading the complement proteins to the active form, which itself has serine protease activity, and degrading downstream molecules in the cascade to the active form. Three cascades of complement activation have been elucidated and named respectively, classical pathway, alternative pathway (AP), and lectin pathway. Although the factors that trigger the cascade and the complement molecules involved are different in each pathway, the activation of complement protein C3 and the subsequent activation of the cascade following C5 share the same pathway. C5 is cleaved into C5a and C5b by the C5 conversion enzyme. C5b binds to the surface of the target cells, then C6, C7, C8, and C9 sequentially bind to C5b to form the membrane attack complex (MAC), which penetrates the lipid bilayer to form channels and destroys target cells. Other complement pathway actions include multiple functions, such as opsonization, phagocytosis mainly caused by C3b, and leukocyte chemotaxis by C5a.

The classical pathway is initiated by the binding of immune complexes (antibodies bound to antigens) to C1q. Thus, it plays a bridging role between acquired and innate immunity.

The lectin pathway is activated by the binding of mannose-binding lectin (MBL) and ficolin to mannose and other sugar chains on the pathogen surface. In contrast to the classical pathway, the lectin pathway does not require antibodies. These two pathways both use a common pathway once initiated: *via* activation of C4, C4bC2a functions as a C3 converting enzyme to initiate the activation pathway below C3. Activation of the two pathways is interrupted when the triggering stimulus is no longer present.

**Figure 1:**
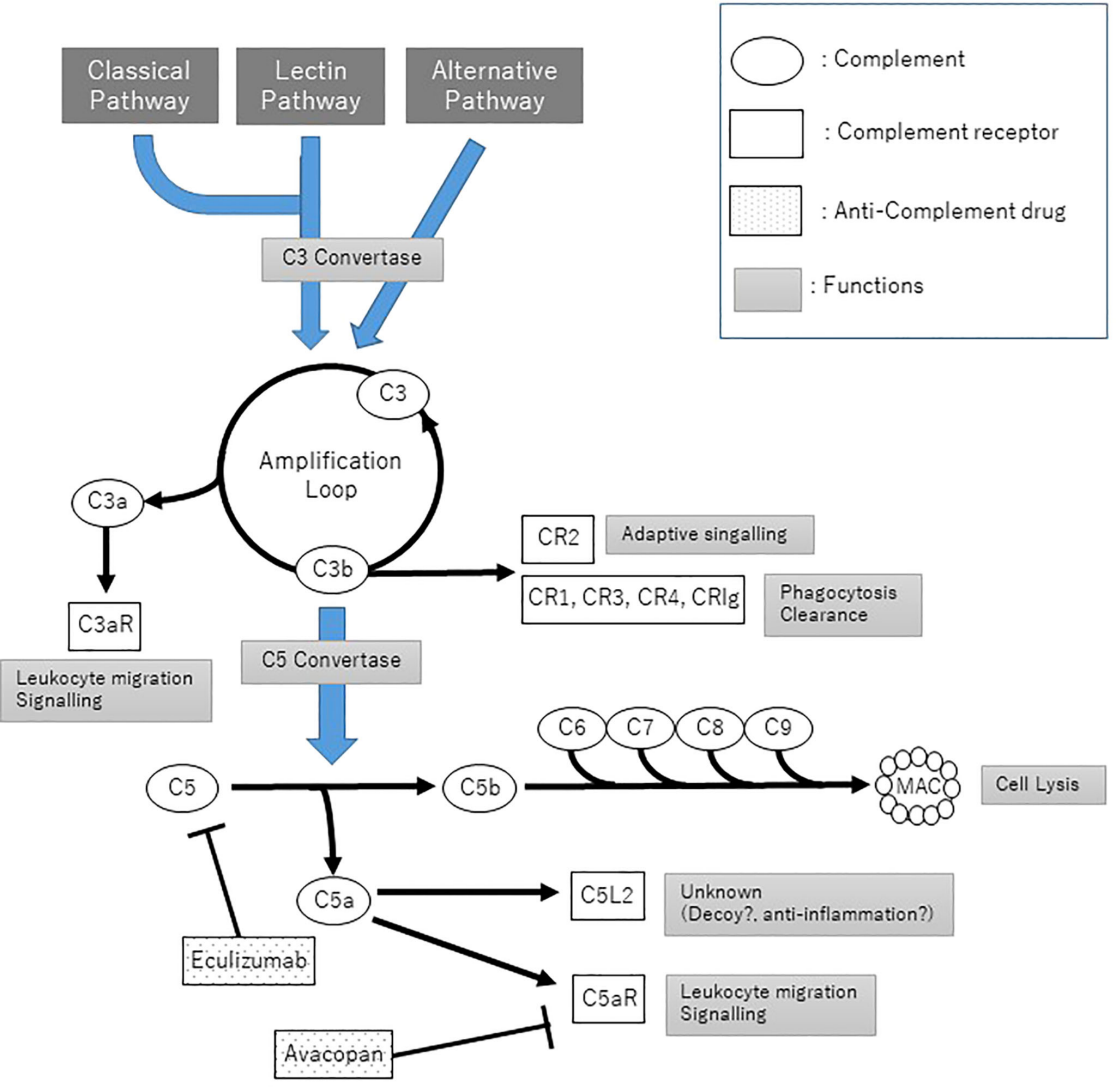
The Complement pathways and anti-complement treatment. C3 convertase: C4bC2a for classical pathway and lectin pathway, C3bBb for alternative pathway; C5 convertase: C4bC2aC3b and C3bBbC3b.

On the other hand, the complemental alternative pathway (cAP) is in a state of constant low-level activation due to C3 hydrolyzation (tick over), and it binds to an activated B factor (C3bBb), which causes activation below C3, similar to C4bC2a in the classical pathway and lectin pathway. The C3b generated as a result of cAP acts as a C3-converting enzyme *via* cAP in a loop to induce conversion to new C3b, leading to amplification of complement activation. Several complement regulatory factors are normally present in the plasma and on the cell surface membrane of the self to prevent excessive C3 activation, but complement activation occurs in the absence of regulatory factors in microbes and foreign substances, thereby resulting in the elimination of the pathogen (**Figure 1**).

## Functions of C5a

In the common pathway for three complement pathways, C5 is degraded into C5a and C5b by C5-converting enzymes (C4bC2aC3b complex in the classical pathway and lectin pathway, C3bBbC3b complex in cAP). C5b leads to the following MAC formation. On the other hand, C5a also induces a variety of immune responses. C5a has two types of receptors, C5aR (CD88) and C5aR2 (C5L2, G protein-coupled receptor 77: GPR77). Both are 7-transmembrane receptors and bind C5a with high affinity. C5aR is expressed on myeloid cells, particularly neutrophils, mast cells/basophils, monocytes/macrophages, and dendritic cells (11); most of the effects of C5a are thought to be mediated by C5aR. At first, C5a was determined as a classical anaphylatoxin, which stimulates the secretion of histamine from mast cells. C5a serves as a powerful chemotactic factor for neutrophils, monocytes, and macrophages, resulting in the migration of these cells to areas where complement activation has occurred (12, 13). C5a supports the survival of neutrophils by delaying the apoptosis (14). C5a also promotes the expression of adhesion molecules on neutrophils. Moreover, C5a causes the production of reactive oxygen species in phagocytes (respiratory burst), activates phagocytosis, and is involved in the degranulation of neutrophils (15). C5a strongly induces neutrophils to release properdin (16), which serves to stabilize C3bBb of cAP, leading to amplification of inflammation by the positive feedback of cAP.

## Complement Alternative Pathway in Animal Models of AAV

The involvement of cAP in AAV was demonstrated using an AAV mouse model that passively transfers MPO-ANCAs. Transfer of antibodies or splenocytes derived from MPO-deficient mice immunized with MPO into wild-type or RAG2-deficient (lacking B and T cells) mice resulted in necrotizing crescentic glomerulonephritis (17). (5-15% mice in wild-type, 80% mice in RAG2-deficient) MPO-ANCA passive transfer into mice lacking C4, which is required for activation of the classical pathway and the lectin pathway of the complement pathway, developed glomerulonephritis as in the wild type. On the other hand, the depletion of C3 using cobra venom factor and mice deficient C5 or factor B completely inhibited the development of glomerulonephritis. Therefore, it is thought that AP plays a central role in the complement pathway involved in AAV. In addition, no suppression of glomerulonephritis was obtained in mice lacking C6, which is involved in MAC, a common downstream component of the three pathways of complement. On the other hand, in C5aR-deficient mice, glomerulonephritis induced by MPO-ANCA passive transfer is completely suppressed, indicating that cAP-mediated C5a and its receptor C5aR are essential for the pathogenesis of glomerulonephritis in the AAV mouse model. Another C5a receptor C5L2-deficient mouse exhibited rather more severe glomerulonephritis than wild-type mice. In addition, the report also showed that the human oral C5a receptor agonist CCX168 dose-dependently suppressed glomerulonephritis in a mouse model of AAV. This suggests that C5a inhibitory therapy may be a therapeutic target (18).

## Complement Alternative Pathway in Humans of AAV (*In Vitro*)

**Figure 2:**
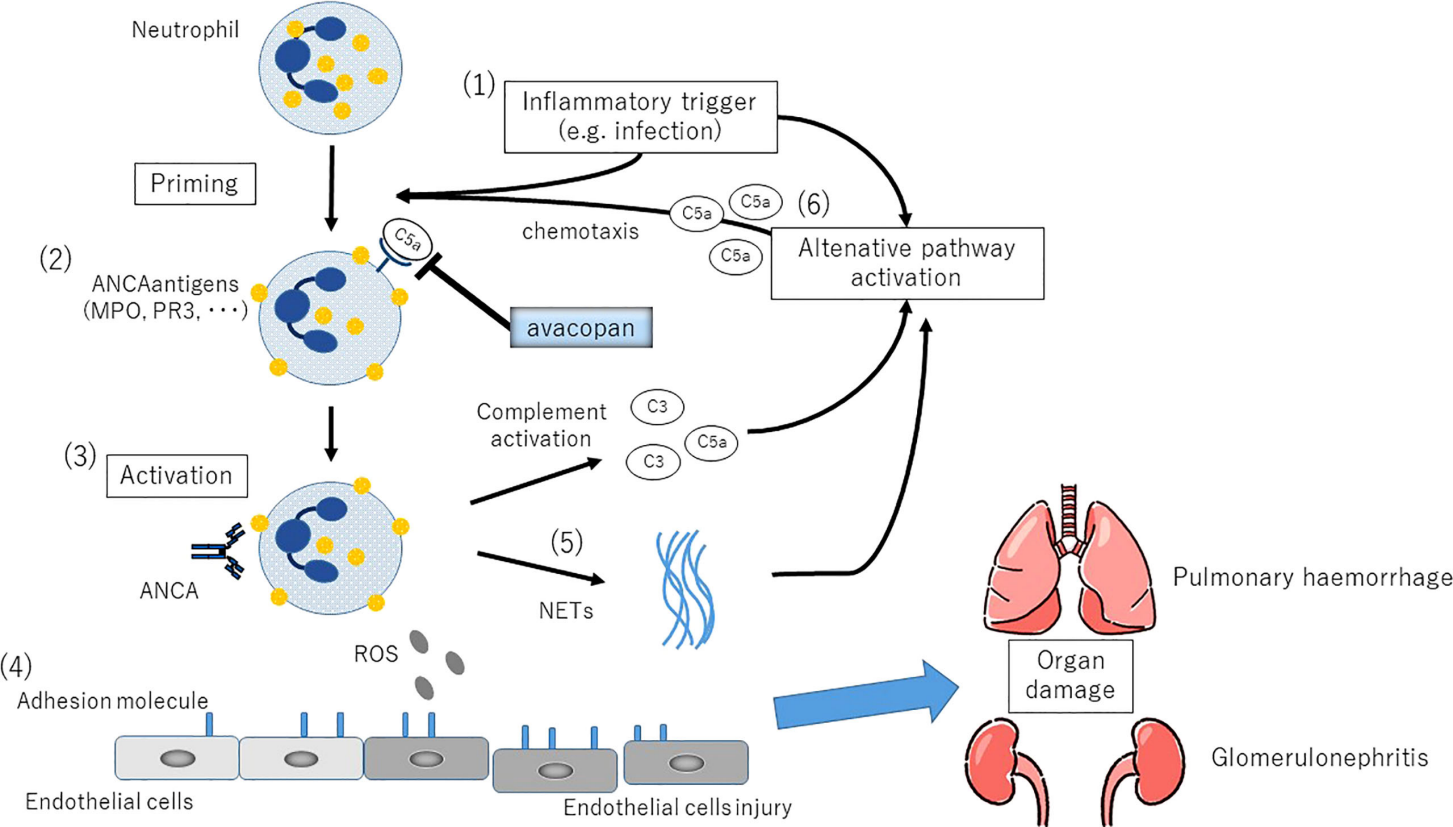
The complemental alternative pathway(cAP) in the etiology of ANCA-associated vasculitis. (1)Inflammatory stimuli such as respiratory tract infections induce neutrophil priming *via* secretion of TNF and other proinflammatory cytokines. (2)Translocation of PR3 and MPO, the counterpart antigens of ANCA, on the neutrophil cytoplasmic membrane. Plasma ANCA becomes accessible to neutrophils. (3)Activation of neutrophils by ANCA. (4)Increased expression of adhesion molecules in small vessels leads to endothelial adhesion, followed by vascular destruction by ROS production and release of degradative enzymes. (5)NETs (neutrophil extracellular traps) production, the persistence of autoantigen presentation, and the production of cAP activating factors. (6)C5a generated as a result of cAP activation promotes local migration and priming of neutrophils, further amplifying this loop.

*In vitro* stimulation of healthy human neutrophils with ANCA isolated from AAV patients generates C3a and C5a in culture supernatants. This response was inhibited only by the C5aR blockade and not by the C3a blockade. It was also shown that C5a, but not C3a, causes ANCA antigen to migrate from the cytoplasm to the plasma membrane, priming neutrophils for the subsequent ANCA-induced respiratory burst (19). It has also been shown that neutrophils primed by cytokines or coagulation factors activate cAPs, which release C5a and amplify the inflammatory reaction (20). It is suggested that these activated neutrophils produce C5a, which amplifies the activation of cAPs, which in turn amplifies the generation of C5a, forming an amplification loop that leads to a prolonged inflammatory condition (21) (**Figure 2**).

## Complement Alternative Pathway in AAV Patients

In patients with active AAV, levels of circulating C3a, C5a, soluble C5b-9, and Bb in the plasma are increased. In contrast, in patients with AAV in remission, soluble C5b-9, and C5a improve after treatment, but C3a levels remain elevated. Plasma C2, C4, and C4d levels which are involved in the classical and lectin pathways are consistently higher in AAV patients than in healthy individuals independent of the disease activity of AAV (22). Multiple reports have linked hypocomplementemia at the time of diagnosis of AAV to diffuse alveolar hemorrhage, skin lesions, thrombotic microangiopathy (TMA), renal failure, and poor survival prognosis (23, 24). Especially in low C3 AAV patients, there was a high frequency of histological TMA findings in renal histology, and many of these patients became refractory to treatment and developed end-stage renal failure (25). On the other hand, serum C4 levels were not associated with AAV prognosis.

Plasma Bb levels, a characteristic marker of cAP activation, were also higher only in patients with active AAV and correlated with the Birmingham Vasculitis Activity Score (BVAS) and pathological findings such as crescentic glomerulonephritis. Decreased levels of circulating properdin, which stabilizes the C3 convertase C3bBb in cAP, have also been reported in AAV patients, and they are inversely correlated with the rate of crescent formation in the glomeruli of the kidney (22).. Urinary C5a was increased in AAV (26), and a correlation was observed between urinary Bb levels and serum creatinine, suggesting that cAP is involved in the development of glomerulonephritis.

The involvement of the complement regulatory factors in AAV has also been investigated. The plasma level of factor H(FH), a major complement regulator of cAP, is significantly lower in patients with active AAV than in healthy subjects and patients with AAV in remission. It has also been shown that plasma levels of the FH are recovered in AAV patients who achieved remission with treatment (27). In addition, abnormalities in the functional activity of the FH are also reported (28). This suggests that cAP activation due to qualitative or quantitative abnormalities in the FH may be related to the development of AAV.

Glomerulonephritis induced by AAV is characterized by immunofluorescence microscopy or immunohistochemical staining, which is typically negative or minimal immunoglobulin deposition, so-called “pauci-immune” glomerulonephritis (29). Afterward, however, it has been reported that in the majority of kidney tissues from AAV patients, immunoglobulin and/or complement components can be detected by electronic microscopy or immunofluorescent examinations (30).

C3d and C5b-9 deposits were found in glomeruli and small vessels in renal histology of patients with active AAV, correlating with cellular crescents formation and proteinuria (31). In a cohort of one hundred twelve AAV patients’ renal biopsies, one-third of the cases showed C3c (C3b cleavage product) deposition, which also correlated with urinary protein levels and renal function (32).

## Complement Alternative Pathway as a Therapeutic Target for AAV

The accumulation of results indicating the involvement of cAP in animal models and *in vitro* studies using human ANCA, as well as in serologic and histologic studies of AAV patients, shown over the years, has led to the concept of cAP as a potential therapeutic target for AAV. Inhibition of C5 itself in AAV may result in loss of the potential anti-inflammatory effect of C5L2-mediated C5a (18). In addition, from the drug safety aspect, direct inhibition of C5 blocks the MAC formation-mediated common pathway of complement activation, which may reduce the innate immune response against microbes, especially encapsulated bacteria such as meningococci. Therefore, C5a seems to be an appropriate target, resulting in a new oral antagonist of C5aR, CCX168 (avacopan; Molecular weight 581). The efficacy of CCX168 on AAV was evaluated in an MPO-induced AAV mouse in which the C5a receptor was replaced by the human C5a receptor (hC5aR mice). CCX168 administration reduced glomerular crescents from 30.4% to 3.3% and decreased neutrophil infiltration into the glomeruli.

## C5a Receptor Antagonist: Avacopan

In a phase I clinical trial of avacopan, forty-eight healthy volunteers received a single dose of avacopan 1, 3, 10, 30, or 100 mg, followed by consecutive doses of 1 mg once daily, 3 mg once daily, 10 mg once daily, 30 mg twice daily, and 50 mg twice daily respectively. It was well tolerated and its pharmacokinetics were dose-dependent. There were no apparent dose-dependent adverse events, which were not associated with serious adverse events and were not significantly different from placebo participants. More than 94% C5aR inhibition was observed with 30 mg twice daily of avacopan (33). Following the phase I trials, the CLEAR study, and the CLASSIC study were conducted as phase II trials.

The CLEAR study (NCT01363388), a double-blind, placebo-controlled trial investigating the efficacy and safety of avacopan in patients with active AAV, was conducted mainly in Europe. The participants were newly diagnosed or relapsed ANCA-associated vasculitis patients, 67 with GPA or MPA (>95% of newly diagnosed patients and with renal involvement) were randomized to CY or RTX followed by AZA in three groups: Group 1: placebo + prednisolone(PSL) 60 mg; Group 2: avacopan 30 mg twice daily + PSL 20 mg; Group 3: avacopan 30 mg twice daily without steroid (34). The dose of PSL was tapered to discontinuation of PSL by 20 weeks for Group 1 and by 14 weeks for Group 2. The primary endpoint was a 50% or greater reduction in BVAS score at 12 weeks, which was achieved in 70%, 86% (p = 0.002 vs. placebo), and 81% (p = 0.01 vs. placebo) of patients in Group 1, Group 2 and Group 3, and in all groups.

The secondary endpoint focused on the response to treatment for glomerulonephritis, the decrease in the urinary albumin/urinary creatinine ratio, was higher in Group 2 (-56% (p<0.01)) and Group 3 (-43%) than in Group 1 (-21%) at Week 12. A marked decrease in urinary protein was observed in the first 2 weeks after the start of avacopan. The other secondary endpoint, the decrease in urinary MCP-1 (monocyte chemoattractant protein-1)/urinary creatinine ratio, was higher in Group 2 (-70%, p<0.001) than in Group 1 (-43%), indicating that the immune response in the local glomerulus has been reduced by avacopan. Adverse events occurred with similar frequency in all groups, with 17% (4/23) of patients in Group 1 and 25% (11/44) of patients in Groups 2 and 3 having serious adverse events. On the other hand, glucocorticoid-related side effects (e.g. psychiatric disorders or glucocorticoid-induced diabetes) tended to be less common in the avacopan-treated group (34% vs. 65%). The CLEAR study showed that avacopan can be an effective and safe alternative to high-dose glucocorticoid treatment in patients with AAV, and in addition, the preliminary urinary findings suggest that avacopan may have an advantage in efficacy compared to conventional treatment.

The CLASSIC study (NCT02222155) is a randomized, double-blind, placebo-controlled, dose-escalation study conducted in North America. In addition to the standard remission induction treatment (RTX or CY and PSL), two doses of avacopan (10 mg or 30 mg, twice daily) were administered (35). Forty-two patients with new or recurrent AAV were assigned to 3 groups: standard therapy + placebo, standard therapy + avacopan 10 mg twice daily, or standard treatment + avacopan 30 mg twice daily. In all patients, PSL was tapered from 60 mg/day to 10 mg/day by week 12. The primary endpoint was the incidence of adverse events, and no significant differences were found in adverse events or serious adverse events. The CLASSIC trial demonstrated the safety of avacopan when added to high-dose corticosteroids and standard treatment for AAV. Although the duration of avacopan administration in these two phase II studies was only three months, they suggest the safety of avacopan (30 mg twice daily) and its potential as a steroid-sparing agent.

**Figure 3:**
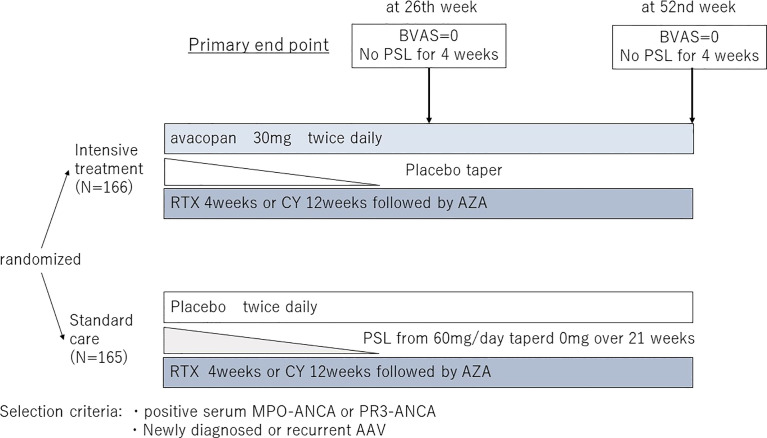
The design of the ADVOCATE trial. BVAS, Birmingham Vasculitis Activity Score; PSL, prednisolone; RTX, Rituximab; CPA, Cyclophosphamide; AZA, Azathioprine; MPO-ANCA, myeloperoxidase-anti neutrophil cytoplasmic antibody; PR3-ANCA, proteinase3-anti neutrophil cytoplasmic antibody.

Following the results of the Phase II study, a large, multicenter, double-blind, double-placebo randomized controlled trial (ADVOCATE study) was conducted as a Phase III study (36) (**Figure 3**). Patients with AAV were randomized 1:1 to either PSL or avacopan in combination with either CY followed by AZA or RTX. One hundred sixty-six patients were included in the avacopan group and one hundred fifty-five in the PSL group. The achievement rate of remission at week 26, the primary endpoint, was 72.3% in the avacopan group versus 70.1% in the PSL group, indicating that avacopan was non-inferior to PSL (p<0.0001), but not superior (P=0.24). Sustained remission at 52 weeks, another primary endpoint, was achieved in 65.7% of the avacopan group and 54.9% of the PSL group, achieving both non-inferiority and superiority of the avacopan group over the PSL group (p=0.0066 for the superiority of avacopan). In addition, eGFR was significantly increased in the avacopan group. Although there was no difference in severe adverse events between the two groups, there was a significant reduction in glucocorticoid-related toxicity in the avacopan group compared to the PSL group. The results were promising not only for the efficacy of avacopan but also for its potential to reduce steroid-related toxicity, which is often a problem in the daily practice of AAV. Safety was also reported to be equivalent in both groups, and the results are encouraging for its usefulness in the real-world clinical setting. As a result, on October 8, 2021, the U.S. Food and Drug Administration (FDA) approved avacopan as adjunctive therapy for adult patients with severe active AAV. Following FDA, avacopan was approved by the Japanese pharmaceuticals and medical devices agency (PMDA) on September 27, 2021, and by the European Medicines Agency (EMA) on January 11, 2022. The regions where the use of avacopan will be available expand in the future. Avacopan is now a new treatment option for AAV used in combination with standard therapy.

## In Closing

Although the complement system was identified at a very early stage in immunological research, there were no drugs modulating complement in clinical practice for a long time. Under such circumstances, the complement system might have been considered clinically less important. On the other hand, subsequent elucidation of the mechanisms of acquired immunity, as well as advances in biological technology, have led to the rapid development in recent decades of novel pharmacological therapies that target cytokines and surface molecules of immune cells. In this context, the involvement of cAP in AAV has been unveiled from mouse models to humans. Inhibition of C5a-mediated neutrophil activation, which is located downstream of the cAP cascade, is considered a novel target for AAV therapy, and the oral C5aR inhibitor avacopan, which has been developed, has demonstrated therapeutic efficacy. A novel agent has just been launched to control inflammatory diseases by regulating the complement system in a so-called “from bench to bedside” approach. It is expected to be a new treatment option that not only controls the disease itself but also optimizes treatment by reducing the side effects associated with corticosteroids. There are still some issues to be explored, such as long-term safety and efficacy, use in induction and maintenance therapy for remission in daily practice, and use in combination with conventional therapies. It is necessary to thoroughly understand the complement system to handle these drugs effectively.

## Author Contributions

YK and TH were involved in drafting the article and approved the final version and the revised version to be published.

All persons who meet authorship criteria are listed as authors, and all authors certify that they have participated sufficiently in the work to take public responsibility for the content.

## Funding

TH reports grants from the Ministry of Health, Labour and Welfare (MHLW) (H29-013), Japan, grants from the Ministry of Education, Culture, Sports, Science, and Technology (MEXT)(16K09246, 19H03564), Japan.

## Conflict of Interest

The authors declare that the research was conducted in the absence of any commercial or financial relationships that could be construed as a potential conflict of interest.

## Publisher’s Note

All claims expressed in this article are solely those of the authors and do not necessarily represent those of their affiliated organizations, or those of the publisher, the editors and the reviewers. Any product that may be evaluated in this article, or claim that may be made by its manufacturer, is not guaranteed or endorsed by the publisher.
